# *Clostridium perfringens* Phospholipase C Induced ROS Production and Cytotoxicity Require PKC, MEK1 and NFκB Activation

**DOI:** 10.1371/journal.pone.0086475

**Published:** 2014-01-23

**Authors:** Laura Monturiol-Gross, Marietta Flores-Díaz, Maria Jose Pineda-Padilla, Ana Cristina Castro-Castro, Alberto Alape-Giron

**Affiliations:** 1 Instituto Clodomiro Picado, Facultad de Microbiología, Universidad de Costa Rica, San José, Costa Rica; 2 Departamento de Bioquímica, Escuela de Medicina, Universidad de Costa Rica, San José, Costa Rica; 3 Centro de investigación en estructuras microscópicas, Universidad de Costa Rica, San José, Costa Rica; II Università di Napoli, Italy

## Abstract

*Clostridium perfringens* phospholipase C (CpPLC), also called α-toxin, is the most toxic extracellular enzyme produced by this bacteria and is essential for virulence in gas gangrene. At lytic concentrations, CpPLC causes membrane disruption, whereas at sublytic concentrations this toxin causes oxidative stress and activates the MEK/ERK pathway, which contributes to its cytotoxic and myotoxic effects. In the present work, the role of PKC, ERK 1/2 and NFκB signalling pathways in ROS generation induced by CpPLC and their contribution to CpPLC-induced cytotoxicity was evaluated. The results demonstrate that CpPLC induces ROS production through PKC, MEK/ERK and NFκB pathways, the latter being activated by the MEK/ERK signalling cascade. Inhibition of either of these signalling pathways prevents CpPLC's cytotoxic effect. In addition, it was demonstrated that NFκB inhibition leads to a significant reduction in the myotoxicity induced by intramuscular injection of CpPLC in mice. Understanding the role of these signalling pathways could lead towards developing rational therapeutic strategies aimed to reduce cell death during a clostridialmyonecrosis.

## Introduction

The anaerobic, spore-forming bacteria *Clostridium perfringens* is the most widely distributed pathogen in nature. It is found in soil, sewage, and in the gastrointestinal tract of humans and many animals [1], [2]. *C. perfringens*-induced myonecrosis, or gas gangrene, is one of the most fulminant Gram-positive infections of humans [3], in which a rapid destruction of viable, healthy tissue occur*s* [4]. Shock and organ failure frequently accompany gas gangrene, and when patients become bacteremic, mortality exceeds 50% [3]. Radical amputation remains the single best life-saving treatment of this disease [4]. *C. perfringens* phospholipase C (CpPLC), also called α-toxin, is an extracellular enzyme produced by all *C. perfringens*strains [5]. Several lines of evidence demonstrate the main role of CpPLC in the pathogenesis of gas gangrene: first, *C. perfringens*mutant strains lacking the plc gene do not cause gas gangrene [6]; second, CpPLC injected intramuscularly causes extensive myonecrosis [7]; and third, immunization with a CpPLC fragment protects mice from a lethal *C. perfringens* infection [8].

CpPLC is a metalloenzymethat hydrolyses mainly phosphatidylcholine and sphingomyelin, although it has a broad substrate specificity [9]. CpPLC induces platelet aggregation, is hemolytic, cytotoxic, myotoxic and lethal [2]. At high concentrations CpPLC causes membrane disruption and cytolysis, but at low concentrations, CpPLC leads to the unregulated generation of second messengers [10]. In intestinal epithelial cells CpPLC activates arachidonic acid metabolism [11]. In rabbit neutrophils, this toxin induces Protein kinase C (PKC) activation and the Mitogen activated protein kinase kinase/Extracellular signal regulated kinase (MEK/ERK) pathway, leading to superoxide production [12]. On a human lung adenocarcinoma epithelial cell line, CpPLC induced production of IL-8, through activation of both an ERK1/2- Nuclear factor kappa B (NFκB) system and a p38 MAPK system [13]. Ganglioside-deficient cells are hypersensitive to CpPLC [14]. Muscle is known to have the lowest concentration of complex gangliosides among mammalian tissues, a condition that could explain the high susceptibility of muscle fibres to the cytotoxic effect of CpPLC[15]. In ganglioside-deficient cells, lytic concentrations (>4 ng/ml) of CpPLC cause membrane damage and lactate dehydrogenase (LDH) release [15], whereas sublytic concentrations (<4 ng/ml) cause oxidative stress and activates the MEK/ERK pathway, which contributes to its cytotoxic and myotoxic effects of this enzyme[16]. However, whether other signalling pathways are involved in CpPLC induced toxicity remains unknown.

Oxidative stress occurs when high concentrations of reactive oxygen species (ROS) deleteriously affect DNA, lipids and proteins of cells. ROS can originate from different sources, including the mitochondrial electron transport chain, xanthine oxidase, myeloperoxidase, nicotinamide adenine dinucleotide phosphate (NADPH) oxidases (NOX enzymes), and lipoxygenase [17] [18]. ROS have been shown to be indispensable for cell survival, differentiation and apoptosis. Various signalling pathways, including MAPKs, NFκB, and PKC, could become activated during ROS generation [19].

Among MAPKs, the MEK-ERK cascade is a central signalling pathway that regulates a wide variety of cellular processes, including proliferation, differentiation, and survival. However, in response to some stimuli, stress response and apoptosis are also induced through ERK 1/2 phosphorilation [20] [21].

NFκB transcription factors regulate the expression of many genes involved in regulating cell growth, differentiation, development, and apoptosis in response to ROS production [18] [22]. Canonical NFκB signalling initiates through phosphorylation of Inhibitor-κB (IκB) by the IκB kinase complex (IKK complex). This triggers IκB degradation, unmasking nuclear localization sequence within the p65/p50 subunits of NFκB, allowing their translocation to the nucleus [22].

The PKC family, which could also be regulated by ROS, is composed of serine/threonine protein kinases involved in a variety of pathways that regulate cell growth, differentiation, apoptosis, transformation and tumorigenicity [17], [23]. Members of the PKC family are classified into three major groups: the classical PKCs, that require calcium, phospholipid and diacylglycerol (DAG) for activation, includes the α, β, and γ isoforms; the novel PKCs, that do not require calcium for activation, includes the θ, η, ε, δ isoforms; and the atypical PKCs, insensitive to DAG, phorbol esters, and calcium, includes the ζ and ι/λ isoforms [24]. Upon activation, PKC isozymes translocate from the soluble to the particulate cell fraction, including cell membrane, nucleus, and mitochondria [23].

The present work aims to clarify the role of ERK 1/2, NFκB and PKC dependent pathways in the cytotoxicity induced by CpPLC.

## Materials and Methods

### Toxin and antibodies

The*C. perfringens* phospholipase C gene from the strain NCTC 8237 was expressed in *Escherichia coli* and the recombinant toxin was then purified as described [7]. The recombinant toxin was used at a sublytic concentration of 3 ng/ml for all experiments using cells. Antibodies used for western blot included: anti PKC βII, anti-clathrin heavy chain from Sigma-Aldrich, anti ERK1/2 pTpY 185/187 and anti ERK 1/2 from Enzo Life Sciences (BiomolInt), anti p50 NFκB from Santa Cruz Biotechnology and anti-phosphoIκB from Abcam.

### Cells and cell culture

Chinese hamster fibroblasts, referred to here as Don Q [15], ormurine melanoma cells, refered to here as GM95 [25], both having a ganglioside deficiency, were cultivated at 37°C in Eagle's minimum essential medium supplemented with 10% fetal bovine serum, 5 mM L-glutamine, penicillin (100 units/ml), and streptomycin (100 mg/ml) in a humid atmosphere containing 5% CO_2_.

### Cytotoxicity assays

Don Q cells in 96-well plates were incubated in the absence or presence of *C. perfringens*CpPLC (3 ng/ml) in 100 µl of medium/well and cell viability was assessed 18 h later using a neutral red assay [15]. Cell survival was expressed as a percentage, considering as 100% the value of parallel cultures incubated with medium and drugs at the indicated concentrations. Assays were performed with 3–6 replicate samples. To evaluate the effect of PKC or NFκB inhibitors in the cell sensitivity to CpPLC, cells were incubated with different drugs in supplemented medium during exposure to serial dilutions of CpPLC. Reagents were obtained from Enzo Life Sciences (BiomolInt) unless otherwise stated. The drugs and the concentrations used for each of them were: GF109203x (5–20 µM); Safingol (7,5–30 µM) (Calbiochem); Hispidin (0,5–2 µM) (Calbiochem); P205 (33–100 µg/ml); P222 (40–80 µg/ml); P219 (33–66 µg/ml); P223 (33–66 µg/ml); Rottlerin (3,6–7,2 µM); helenalin (0.5–1 µg/ml), BAY 11-7085 (0.66–10 µg/ml), CAPE (4 µM–20 µM), clasto-lactacystin β-Lactone (6–10 µM); NFκB SN50 (P605, 33–133 µg/ml), IKK NBD (P607, 33–133 µg/ml). These substances were not cytotoxic at the concentrations used (viability was higher than 90% in cells exposed only to the drugs in comparison with untreated cells).

### Myotoxicity assays

Mice were handled according to protocols approved by the Institutional Committee for Care and Handling of Experimental Animals of the Universidad de Costa Rica. Groups of 10 CD-1 mice of 16–18 g were injected in the right gastrocnemius with 1.1 µg of CpPLC. The creatine kinase (CK) activity in plasma was determined using the CK-10 assay (Sigma) [7] Mice received intraperitonealy 100 µl of phosphate-buffered saline (PBS) (Control), BAY 117085 (BAY, 20 µg) or Helenalin (3 µg) in 100 µL of PBS 1 hour before and 1 hour after toxin challenge. None of the substances had evident toxic effects in mice at the doses used, which were chosen according to the references.

### PKC activity

Don Q cells were exposed to each drug overnight and later treated with CpPLC (3 ng/ml), or with Phorbol 12 myristate 13 acetate (PMA) (Abcam) as positive control. PKC activation was determined using Promega'sPepTag® Non-Radioactive Protein Kinase C Assay, according to manufacter instructions. Briefly, cells were washed after each treatment with PBS, and suspended in cold PKC extraction buffer, homogenized, centrifuged and supernatant was passed over a 1 ml column of DEAE cellulose. PKC containing fraction was eluted and its activity towards PepTag® C1 peptide was assessed and later visualized in agarose gel electrophoresis as phosphorilated peptide (negatively charged) migrates towards the cathode.

### Reactive oxygen species (ROS) production

Don Q cells were treated overnight with each inhibitor, and after 7 h of incubation in the absence or presence of CpPLC (3 ng/ml) at 37°C, Don Q cells were loaded with 2′7′-dichlorodihydrofluorescein diacetate (DCFDA) (5 µM) for 30 min, washed with PBS, tripsinized and propidium iodine stained. A FACSCalibur flow cytometer (Becton Dickinson) was immediately used to collect data for 5×10^3^ cells and analysis was performed using CELLQUEST (Becton Dickinson) to determine intensity and percentage of 2′7′-dichlorodihydrofluorescein (DCF) fluorescent cells, formed by oxidation with intracellular ROS, among live cells.

### Western blots

For PKC βII detection, Don Q cells were exposed to CpPLC (3 ng/ml) for different time periods and cytosol and membrane fractions were separated according to Abcam subcellular fractionation protocol (provided by Dr. Richard Patten, Tufts New England Medical Center, MA). Equal amount of protein of the membrane or cytosol fraction were electrophoresed and transferred (SDS-PAGE 4–20%). PKC βII was detected in both fractions and membranes were stripped and used for clathrin (membrane fraction) or actin (cytosol fraction) detection. For ERK or phosphoIkB detection, Don Q cells were pretreated overnight with inhibitors before and during CpPLC (3 ng/ml) exposure. Cells were then resuspended in NP-40 lysis buffer. Equal amounts of total protein were electrophoresed (SDS-PAGE 12%) and transferred. Phosphorylated ERK or phosphoIκB were detected and membranes were stripped and used for total ERK or actin detection. For p50 NFκB detection, Don Q cells were exposed to CpPLC for different time periods and nuclear fraction was separated as previously described [26]. Equal amount of protein of the nuclear fraction was electrophoresed and transferred. Chemiluminescence substrate (Invitrogen) was used to develop the reaction. Images were taken with Chemidoc XRS TM imaging system (BioRad) and bands of each protein of interest were quantified vs loading control using Image J software and expressed as relative density % of negative control.

### Statistical analysis of data

Statistical significance was determined by ANOVA and post-hoc Tukey test, or by t-student test, using STATISTICA vs 6.0 software.

## Results

### Protein kinase C activation is required for CpPLC cytotoxicity

**Figure 1:**
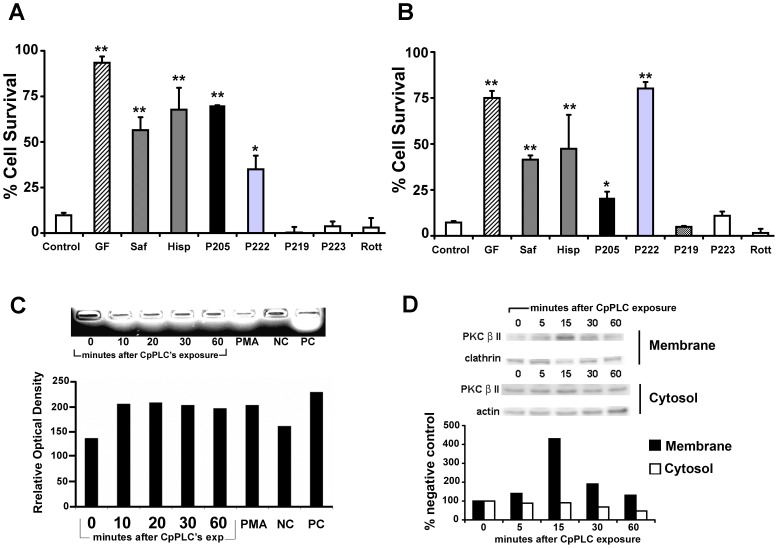
PKC activation is required for the cytotoxic effect of CpPLC. Don Q cells (A) or GM95 cells (B) were treated overnight with GF109203x (GF, 20 µM (A), 10 µM (B)); Safingol (Saf, 10 µM (A), 30 µM (B)); Hispidin (His, 2 µM); P205 (66 µg/ml (A), 100 µg/ml (B) P222 (40 µg/ml); P219 (66 µg/ml); P223 (66 µg/ml) and Rottlerin (Rott, 7,2 µM) or MEM (control) before exposure to CpPLC. Cell viability was determined 18 h later using the neutral red assay. Results are expressed as the percentage of neutral red incorporated by the remaining cells, in comparison with the neutral red incorporated by control cells incubated with each treatment, but not exposed to the toxin. The results represent the average of two-four independent experiments with three replicate samples. (** p<0.001, * p<0.01). (C) Don Q cells were treated without (0 minutes) or with CpPLC for 10, 20, 30 or 60 minutes at 37°C. PMA was used as positive control for PKC activation. Cells were collected and PKC activation was measured using Promega'sPepTag® Non-Radioactive Protein Kinase Assays, according to manufacturer instructions. Results are representative of three independent experiments. (D) Don Q cells were treated without (0 minutes) or with CpPLC (3 ng/ml) for 5, 15, 30 or 60 minutes. Cytosol and membrane fractions were separated as described in the Materials and Methods section, and immunoblotted against PKCβII. After stripping of the blot, actin or clathrin were also detected. Densitometric analysis was performed using Image J software. Results are representative of three independent experiments.

CpPLC induces the activation of PKC on rabbit neutrophils, which participates in superoxide production [12]. Activation of PKC by CpPLC was also detected in Don Q cells (Fig 1C), by detecting its enzymatic activity towards a synthetic peptide. To assess the role of PKC activation on CpPLC induced cytotoxicity, the effect of several PKC inhibitors on Don Q and a parallel cell line that is also ganglioside deficient, GM95, was evaluated (Fig 1A and B). Results showed that PKC inhibitors GF109203X [27], safingol ([(2S,3S)-2-amino-1,3-octadecanediol]) [28], [29], and Hispidin[30] diminished the cytotoxic effect of CpPLC, clearly involving PKC in CpPLC's mechanism of action. To discern which isoform of PKC is involved, cells were treated with myristoylated PKC peptides that inhibit specific PKC isoforms; P205, a PKCα and β inhibitor [31], [32], and P222, a PKC α, βI, βII and γ inhibitor [31], [33] exert a protective role towards CpPLC. On the contrary, P219, a PKC ζ and γ inhibitor [31], [32], [34], [35]; P223, a PKCε inhibitor [31], [33], [36] and Rottlerin, a PKCδ inhibitor [37], [38] did not have any effect on CpPLC 's cytotoxicity, suggesting that the classical PKCs α and β are the main PKCs involved in CpPLC's mechanism in both cell lines. Furthermore, after a membrane and cytosol separation of Don Q cells, treated with or without CpPLC, both an increase in PKCβ at the membrane fraction, as well as a decrease in PKCβ cytosolic fraction, could be observed due to its membrane translocation after activation (Fig 1D).

### ERK1/2 activation induced by CpPLC is independent of PKC and ROS

**Figure 2:**
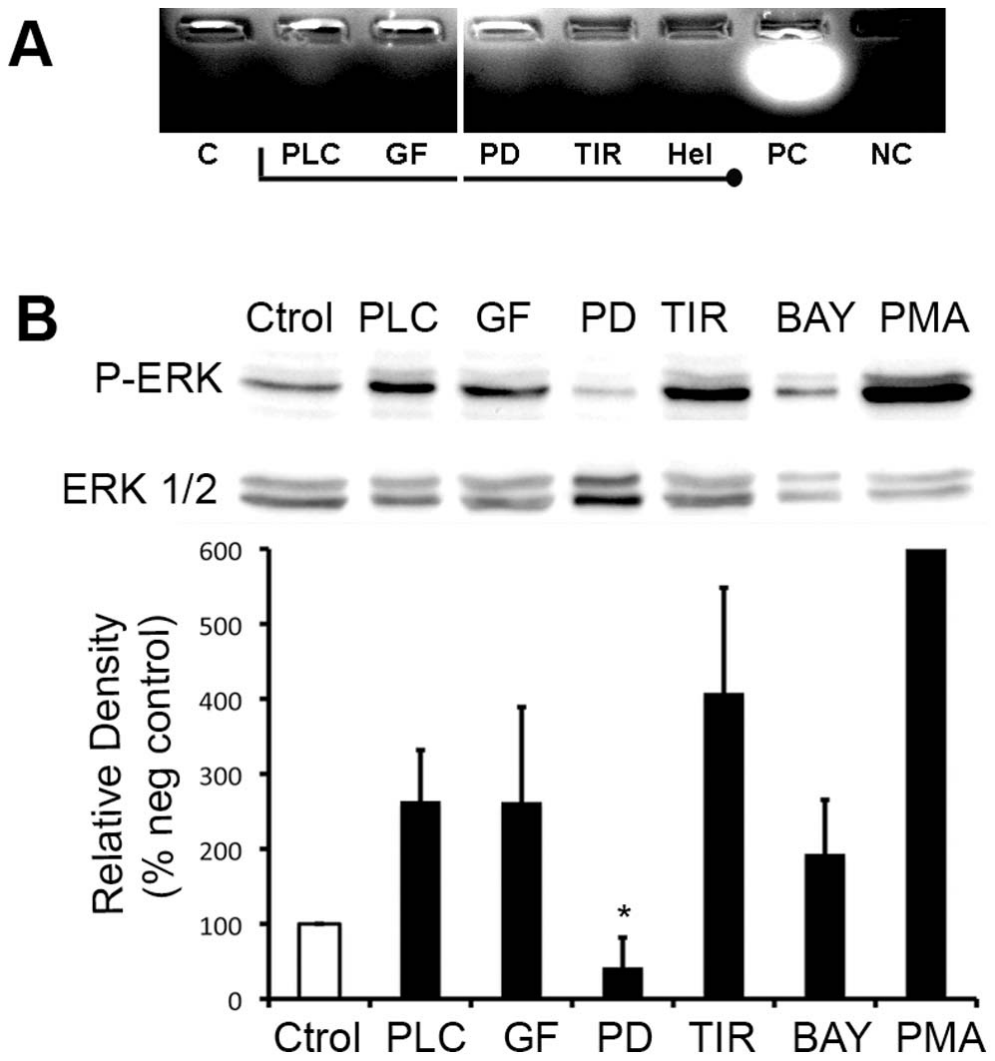
ERK1/2 becomes activated independently of PKC and ROS on Don Q cells exposed to CpPLC. (A) Don Q cells were treated overnight with GF109203x (GF, 20 µM), 2-Aminoethoxydiphenyl borate (2APB, 70 µM) PD98059 (PD, 187 µM), Tiron (TIR, 2 mg/ml) or Helenalin (Hel, 1 µg/ml), previously to CpPLC (PLC) exposure (PC: positive control, NC: negative control, C: control of cells without CpPLC). Cells were collected and PKC activation was measured using Promega'sPepTag® Non-Radioactive Protein Kinase Assays, according to manufacturer instructions. Results are representative of two independent experiments. (B) Don Q cells were treated overnight with GF109203x (GF, 20 µM), PD98059 (PD, 187 µM), Tiron (TIR, 2 mg/ml) or BAY117085 (BAY, 0,004 µg/µl) or PMA (100 nM) as positive control (Y value = 1451±1095), then cells were lysed and immunoblot-ted against phospho-ERK. After stripping of the blot, total ERK1/2 was also evaluated. Densitometric analysis was determined using Image J software. Results are representative of five independent experiments (mean±SD) (*p<0,001 vs PLC).

To test the effect of different pathways over PKC activation by CpPLC, Don Q cells were treated with different inhibitors before CpPLC's treatment. Neither 2-(2′-amino-3′-methoxyphenyl)-oxanaphthalen-4-one (PD98059), a MEK/ERK inhibitor; the superoxide scavenger Tiron or Helenalin, which alkylates p65 NFκB; had any effect on PKCs activation by CpPLC on Don Q cells (Fig 2A). To test the effect of PKC, NFκB or ROS over ERK1/2 activation by CpPLC, Don Q cells were treated with the PKC inhibitor GF109203X, 3-(4-t-butylphenylsulfonyl)-2-propenitrile (BAY), which blocks IκB degradation, and superoxide scavenger Tiron (Fig 2B) before exposure to CpPLC. PD98059, a ERK 1/2 inhibitor was used as control. ERK1/2 activation was then evaluated by western blot detecting ERK 1/2 phosphorylation. Results demonstrate that ERK 1/2 activation occurs independently of PKC, superoxide or NFκB, given the results with GF, Tiron or BAY respectively.

### NFκB activation is involved in CpPLC's cytotoxicity and myotoxicity

**Figure 3:**
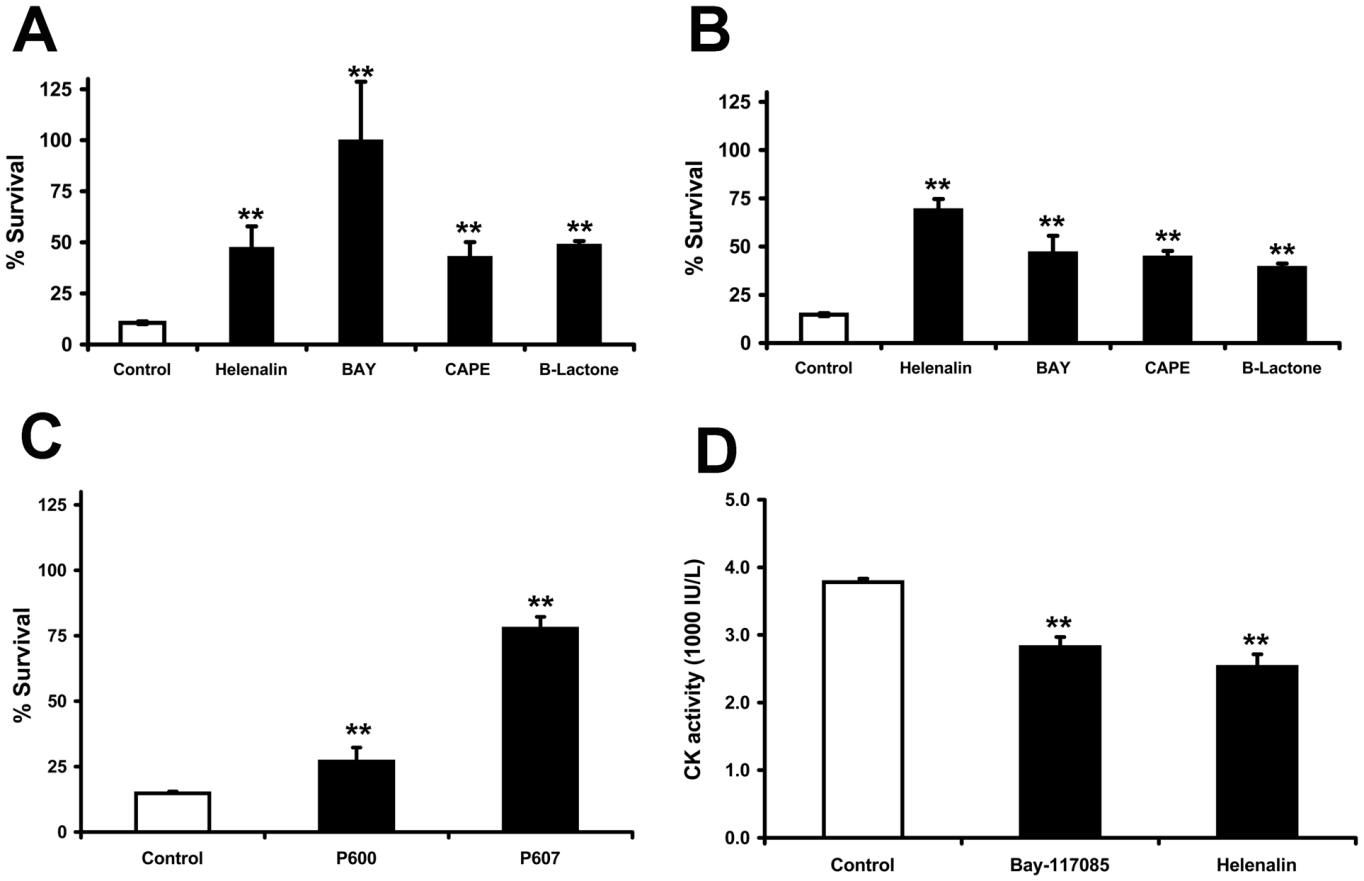
NF-κB activation is required for the cytotoxic and myotoxic effects of CpPLC. Don Q cells (**A**) or GM95 cells (**B**) were preincubated overnight with helenalin (1 µg/ml (A), 0.5 µg/ml (B)), BAY 11-7085 (BAY, 5 µg/ml (A), 0.66 µg/ml (B)), CAPE (10 µM (A), 4 µM (B)), β-Lactone (3 µM (A), 5 µM (B)) or MEM (Control) before exposure to CpPLC(**C**) GM95 cells were exposed to NF-κB SN50 (P600, 133 µg/ml), IKK-NBD (P607, 133 µg/ml) or MEM (Control) overnight before exposure to CpPLC. (A, B, C) Cell viability was determined 18 h later using the neutral red assay. Results are expressed as the percentage of neutral red incorporated by the remaining cells in comparison with the neutral red incorporated by control cells incubated with each treatment, but not exposed to the toxin. The results represent the average of two-four independent experiments with three replicate samples (mean±SE, ** p<0.001). (**D**) Groups of 10CD-1 mice were challenged intramuscularly with 1.1 µg of CpPLC, and after 3 hours, creatin kinase (CK) activity was measured in plasma. One hour before and one hour after toxin injection, mice received intraperitonealy 100 µl of PBS (Control), BAY 117085 (BAY, 20 µg) or Helenalin (3 µg). Results are means±SE of 2–3 independent experiments. (**p≤0,001).

The influence of NFκB in CpPLC-induced cytotoxicity was determined by evaluating the effect of several NFκB inhibitors (Fig 3 A, B, C). Both BAY and clasto-lactacystinβ-Lactone block IκB degradation, inhibiting IκB phosphorylation and the proteasome, respectively; caffeic acid phenethyl ester (CAPE) inhibits NFκB nuclear translocation, and helenalin blocks DNA binding by alkylating p65. These four inhibitors significantly reduced cell death (Fig 3A and B), indicating that NFκB activation is indeed involved in CpPLC-induced cytotoxicity. Furthermore, the effect of two cell permeable peptides which inhibit NFκB activation was also evaluated (Fig. 3C). The IKK-NBP peptide (P607) inhibits IκB kinase, whereas SN50 (P605) inhibits NFκB p50 translocation into the nucleus. Both peptides significantly reduced CpPLC-induced cell death.

NFκB contributes to the injury caused by oxidative stress in various tissues [39], [40]; therefore, NFκB was investigated to determine if its inhibition can alleviate the myotoxic effect of CpPLC. Muscle damage induced by CpPLC was significantly reduced in mice treated with BAY or Helenalin, in comparison with that induced in untreated animals (Fig 3D), thus indicating that NFκB activation contributed to CpPLC's induced myonecrosis.

### NFkB activation depends on ERK1/2 in Don Q cells treated with CpPLC

**Figure 4.CpPLC:**
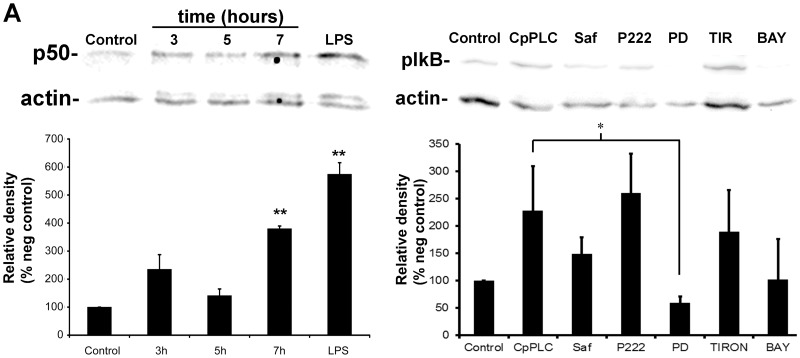
activates NFκB in Don Q cells. (A) Don Q cells were treated without (control) or with CpPLC at different times (3, 5 or 7 hours), and separation of nuclear fraction was performed. Equal amount of protein in the nuclear fraction was electrophoresed and transfered. NFκB p50 was detected, followed by stripping and actin detection (with remaining of p50 just above actin band). Results represent three independent experiments (mean ± SE) (**p<0,01 vs control) (B) Don Q cells were treated overnight with Safingol (Saf, 10 µM), P222 (40 µg/ml), PD98059 (PD, 187 µM), Tiron (TIR, 2 mg/ml), or BAY117085 (BAY, 0,004 µg/µl), and then treated with or without CpPLC (Control) for 7 hours. Cells were lysed and immunoblotted against phospho-IκB. After stripping of the blot, actin was also detected. Densitometric analysis was determined using Image J software. Results represent three independent experiments (mean ± SE) (*p<0,05).

To verify that CpPLC activates NFκB, Don Q cells were treated for various times with the toxin, and nuclear fraction of the cells were separated to detect p50 nuclear translocation. NFκBp50 translocation towards nucleus can already be seen 3 hours after CpPLC's treatment, reaching its highest level after 7 hours (Fig. 4A). NFκB activation at 7 hours was confirmed by detecting phospho-IκB in Don Q cells treated with CpPLC (Fig 4B). PD98059, the MEK1 inhibitor, impairs IκB phosphorylation, suggesting that NFκB activation is dependent of ERK1/2. On the contrary, Safingol, a PKC inhibitor [28] [41]; P222 a PKC α, βI, βII and γ inhibitor; or Tiron, a superoxide scavenger that protects against CpPLC toxic effect [16] did not have a significant effect over IκB phosphorylation by CpPLC.

### ROS generation by CpPLC is dependent of PKC, MEK1 and NFκB

**Figure 5.PKC,:**
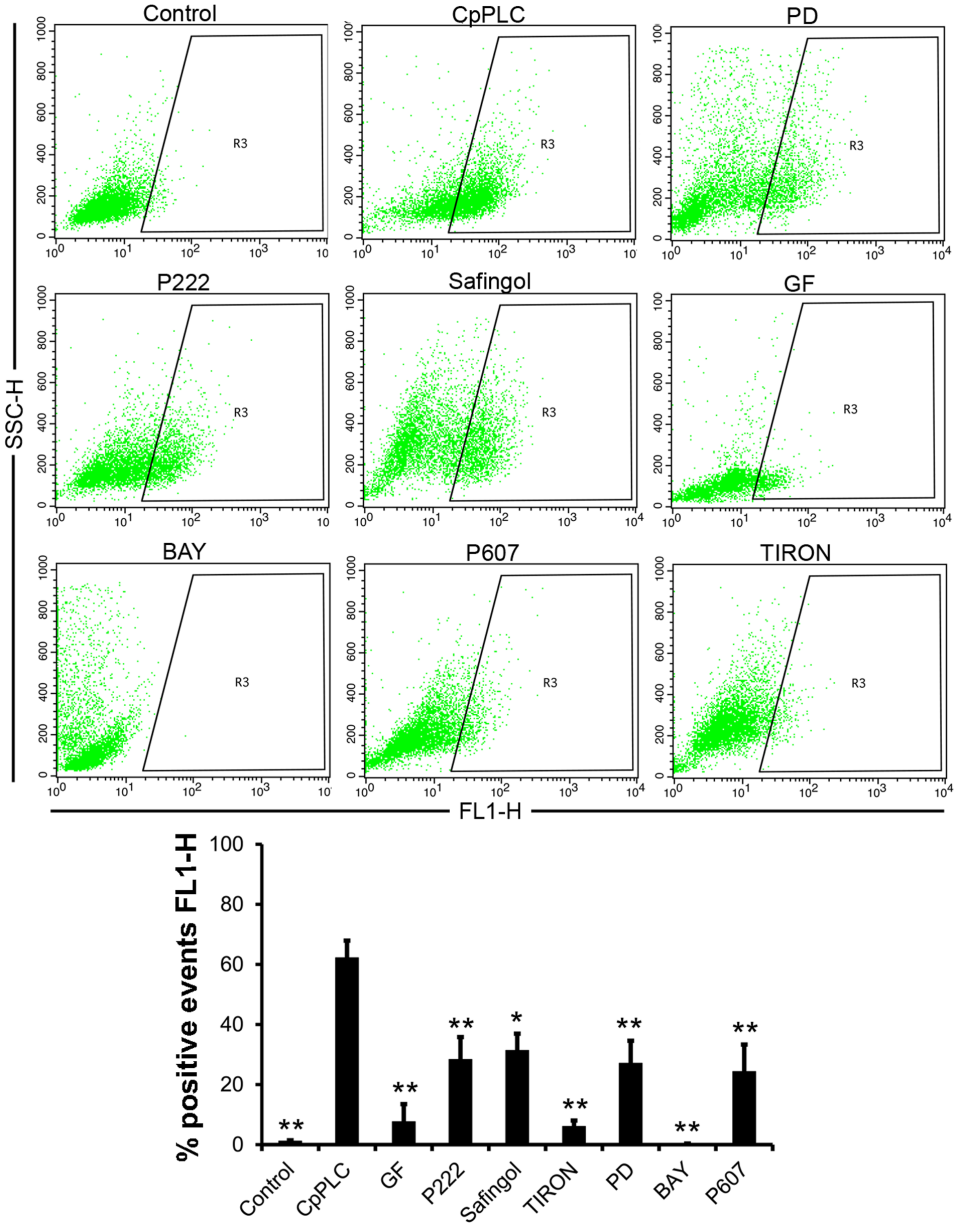
MEK1 and NFκB are required for CpPLC's ROS production in Don Q cells. Don Q cells were treated overnight with GF109203x (GF, 20 µM), P222 (40 µg/ml), Safingol (15 µM), PD98059 (PD, 187 µM), Tiron (2 mg/ml), BAY117085 (BAY, 0,004 µg/µl) or P607 (66 µg/ml) prior to a 7 hour exposure to CpPLC. Cells were then loaded with DCFDA (5 µM) for 30 min, washed with PBS, tripsinized and propidium iodine stained. A FACSCalibur flow cytometer (Becton Dickinson) was immediately used to collect data for 5×10^3^ cells, and analysis was performed using CELLQUEST (Becton and Dickinson) to determine intensity and percentage of DCF fluorescent cells (FL1-H) among living cells. Results are representative of 3–4 independent experiments (mean ± SE) (**p<0,001; *p<0,05).

CpPLC exerts its cytotoxic and myotoxic effect through ROS generation [16]. To clarify the role of PKC, MEK1 and NFκB pathways over ROS production, Don Q cells were treated with different inhibitors of these pathways, and then with CpPLC (Fig 5). ROS production was evaluated using the cell permeable probe DCFDA. ROS accumulation in cells exposed to CpPLC was preventable in the presence of three PKC inhibitors, Safingol, GF and P222 as well as by the MEK inhibitor PD98059. Furthermore, two NFκB inhibitors, BAY and the cell permeable peptide P607, impair ROS production in Don Q cells exposed to CpPLC. Taken together, all the results clearly demonstrate that PKC, MEK1 and the NFκB pathways lead towards ROS generation and the cytotoxic effect caused by CpPLC.

## Discussion

**Figure 6:**
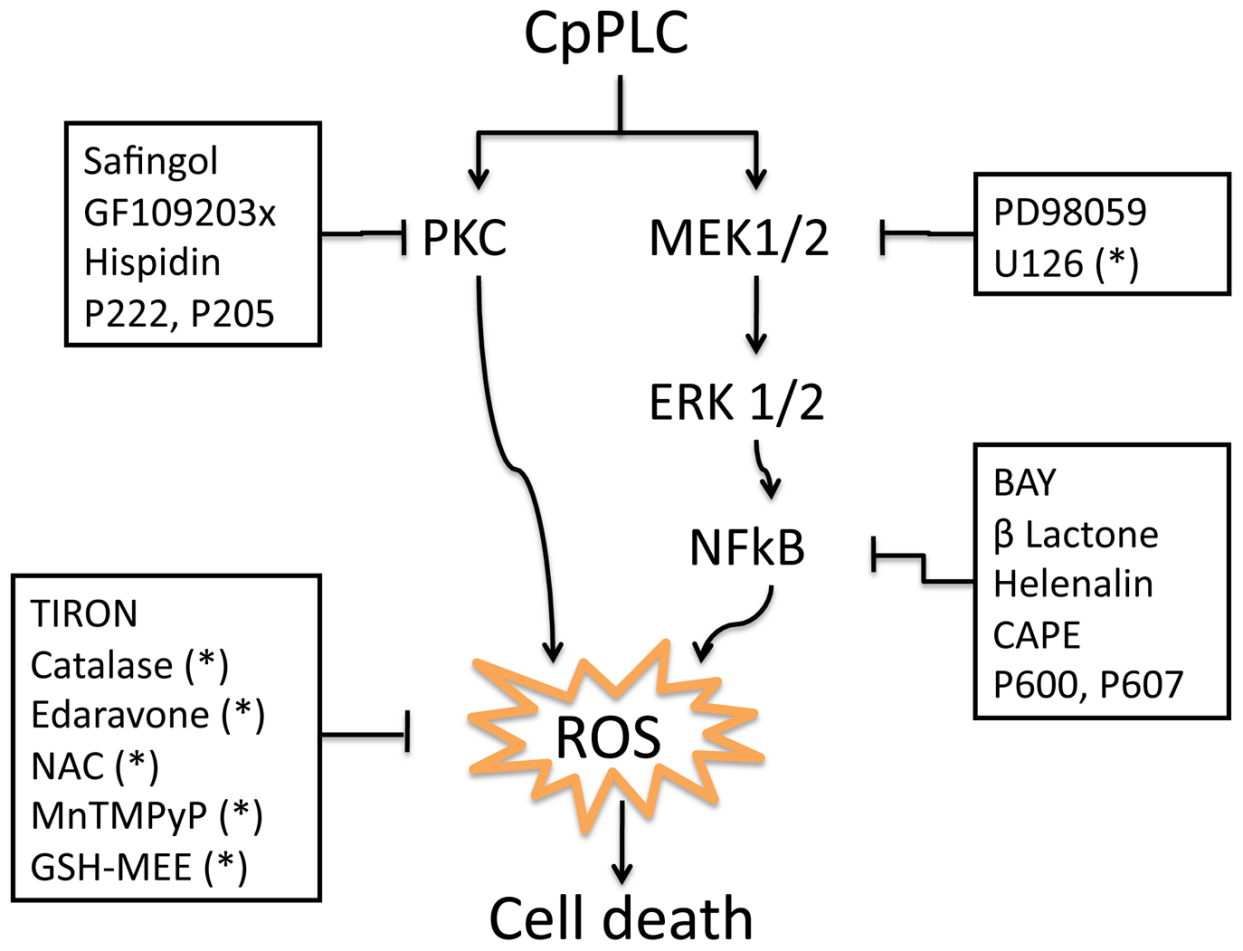
Proposed model describing the mechanism of action of CpPLC leading towards cytotoxicity on ganglioside-deficient cells Don Q. The present study suggests a model where CpPLC activates PKC, MEK/ERK-1/2, and NFκB pathways. MEK/ERK pathway activates NFκB. Inhibition of both ERK-NFκB and PKC pathways inhibit ROS production and cytotoxicity by CpPLC in Don Q cells. (*) Results previously published [16]. Abbreviations: N-acetyl-L-cysteine(NAC); manganese (III) tetrakis (1-methyl-4-pyridyl) porphyrinpenta- chloride (MnTMPyP); glutathione monoethyl ester (GSH-MEE).

CpPLC is a prototype phospholipase C from a group of enzymes which have hydrolytical activity against phosphatidylcholine and sphingomyelin, that also includes LmPLC from *Listeria monocytogenes* and PicHR_2_ from *Pseudomonas aeruginosa* [42]. It has been proposed that, at low doses, CpPLC causes limited phospholipid hydrolysis in the target plasma membrane, thus activating DAG- and ceramide-mediated signal transduction pathways which lead to the uncontrolled production of several intracellular mediators [10], [43]. At sublytic concentrations on ganglioside-deficient cells, this toxin induces ROS generation and activates the MEK/ERK pathway which are important for its cytotoxic and myotoxic effect [16]. However, CpPLC downstream signalling in these cells is not completely understood. The present study demonstrates that CpPLC induces ROS production through PKC, MEK/ERK and NFκB pathways, the latter being activated by a MEK/ERK signalling cascade. It also implicates all these intracellular pathways directly with CpPLC's cytotoxic effect (Fig. 6).

Since Titball's proposal of a PKC activation due to DAG generation by bacterial phospholipases C [44], CpPLC generated PKC activity has been demonstrated on three different systems: rabbit erythrocytes [45], rabbit neutrophils [12], [46] and MDCK cells [47]. Accordingly, PKC activation was also demonstrated on Don Q cells as soon as 10 minutes after CpPLC's treatment. Furthermore, several PKC inhibitors improved survival of ganglioside-deficient cells towards CpPLC, clearly involving PKC in toxin's cytotoxic effect. Among these inhibitors, myristoylated PKC peptides, that inhibit specifically α, β and γ PKC isoforms, impaired cytotoxicity induced by CpPLC, and PKC β activation was detected by western blot of Don Q cells; suggesting the involvement of classical PKC isoforms on CpPLC's mechanism of action. Although a PKCθ, a novel PKC, was previously found to be responsible of generating superoxide anion on rabbit neutrophils [12], many ROS effects and interactions appear to be cell type-specific [18]. Differences could be explained by the use of a sublytic dose, later exposure times of CpPLC to detect PKC activation in the present study, and the use of a different cell system. Safingol is known as a PKC inhibitor which acts on the PKC regulatory domain displacing its ligand from the lipid binding site [48]; GF inhibits PKC activity exclusively via its ATP-binding site [27], [49], and P222 is a peptide derived from the binding site of PKCβII to its anchored protein RACK1, highly conserved among C2 region of classical PKCs. These three drugs inhibited CpPLC's ROS generation on Don Q cells. Previous research show that the activation of the PKC/Noxsignaling complex regulates ROS levels and is involved in various pathophysiological conditions [17], also related to oxidative stress, such as Parkinson disease [50], atherosclerosis [51] and hypertension [52]. The β isoform of PKC has been shown to induce ROS generation as well, through mitochondrial damage [53], [54]. Thus, whether PKC activation by CpPLC could be triggering ROS production by these pathways has to be assessed in future studies.

ROS have been reported to activate ERK1/2 during cell death in different systems [55]–[59]. MEK1-ERK1/2 activation, which occured soon after CpPLC treatment, was previously found to be important for CpPLC's cytotoxic effect on ganglioside-deficient cells. However, downstream signalling leading toward ROS generation was unclear. Results demonstrated that ERK1/2 activation occurs independently of PKC and ROS. Furthermore, it was found that MEK-ERK participates on ROS production in Don Q cells. The ERK1/2 family of protein kinases participates in a wide variety of cellular processes, and the 90 kDa ribosomal S6 kinase (RSK) family are key components downstream from the MEK-ERK signalling cascade. The RSK family regulates transcription by mediating the phosphorylation of a number of transcription factors including NFκB [60].

There are many different potential intracellular sources of ROS, several of which are capable of influencing, or being influenced by NFκB activity [18]. Most commonly, NFκB activation inhibits programmed cell death; however, NFκB may promote cell death under certain circumstances. In response to several oxidative stimuli such as ultraviolet light and chemotherapeutics like doxorubicin or etoposide, NFκB triggers apoptosis [60]. In the present study, NFκB activation by CpPLC was demonstrated on Don Q cells. Furthermore, NFκB inhibition reduces the cytotoxic effect of CpPLC, showing its involvement in cell death. Since the inhibitors used act through different mechanisms, it is unlikely that their protective effect results from non-specific alterations. NFκB activation on Don Q cells was shown to depend on the MEK/ERK pathway. NFκB controls the transcription of several proinflamatory genes, whose overexpression in vivo could contribute to tissue injury. A previous study demonstrated that CpPLC induces IL-8 production in cultured cells by a ERK1/2-NFκB pathway as well [13]; thus, this cytokine, which is a potent neutrophil chemoattrachtant, is potentially involved in gas gangrene pathophysiology.

Treating Don Q cells with Bay or P607, two NFκB inhibitors, ROS generation by CpPLC was impaired. NFκB activity can influence ROS levels via increased expression of antioxidant proteins, such as manganese superoxide dismutase, catalase and glutathione peroxidase. However, NFκB can also trigger the production of ROS by inducing the expression of pro-oxidant targets such as NAD(P)H oxidase Nox 2 [61], xanthine oxidoreductase, cyclooxygenase-2 (Cox-2), inducible nitric oxide synthase (iNOS), lipoxygenases (LOX) and cytochrome p450 enzymes [18]. These are possibly ways by which NFκB produces ROS in cells treated with CpPLC.

NFκB activation is associated with several conditions in which there is muscular damage. NFκB plays a role in the pathogenesis of muscle damage during ischemia/reperfusion such as during a myocardial infarction and cerebral ischemia; and is activated in response to conditions that cause muscle loss [38], [39], [62]–[64]. In patients with Duchene Muscular Dystrophy, the activity of NFκB in the muscle is increased; and, interventions that reduce NFκB in Duchene Muscular Dystrophy models significantly reduce damage and pathology [65]. Remarkably, NFκB inhibition diminishes the myotoxic effect of CpPLC, suggesting that NFκB may be another target for development of therapeutic strategies to reduce tissue damage during gas gangrene.

Overall the results of this work demonstrate that CpPLC induces activation of PKC and MEK/ERK-NFκB pathways in ganglioside deficient cells. These pathways lead towards ROS production and are involved in CpPLC's cytotoxic effect. In addition a reduction in CpPLC's induced myotoxicity was demonstrated by inhibition of NFκB in mice. Understanding the molecular basis of CpPLC induced cell death could lead to rational therapeutic strategies that benefit patients with gas gangrene.
